# Supplementary material for: Identifying Patients with Bicuspid Aortic Valve Disease in UK Primary Care: A Case–Control Study and Prediction Model
Source: J Pers Med. 2022 Aug 5;12(8):1290. doi: 10.3390/jpm12081290 (PMC9410317; doi:10.3390/jpm12081290)
Supplement: Supplementary file 1 [file jpm-12-01290-s001.zip › jpm-1852703-supplementary.pdf]

Supplementary Files

Supplementary material S1: Variables used for imputation.

To estimate missing values for blood pressure, pulse rate, BMI, multiple imputation with chained equations was used based on the following variables were used.

BMI, Diastolic BP, Systolic BP, Mean pulse, mean potassium, mean calcium, gender, diagnosis of hypertension, bradycardia diagnosis, tachycardia diagnosis, ethnicity, IBS diagnosis, atrial fibrillation diagnosis, stroke diagnosis, palpitation diagnosis, collapse diagnosis, betablocker category

Supplementary material S2. Continuous variables missing data

Variable	**Missing data cases (2898) n (%)**	**Missing data controls (14,487) n (%)**
BMI	1089 (37.6%)	4737 (32.7%)
BP	58 (2.0%)	1393 (9.6%)
Pulse	966 (33.3%)	6372 (44.0%)

Supplementary material S3. Additional iterations of model with AUC, AIC and BIC.

Clinical Variable	Model 1	Model 2	Model 3	Model 4	Model 5	Model 6	Model 7	Model *8*
Diagnosis Hypertension	x	x	x	x	x	x	x	x
Diagnosis of tachycardia			x	x	x	x		x
Beta blocker category	x	x	x	x	x	x	x	x
Diagnosis atrial fibrillation (AF)	x	x	x	x	x	x	x	x
Diagnosis palpitations	x	x	x	x	x	x		x
Diagnosis Heart failure					x			
Diagnosis peripheral arterial disease					x			
Diagnosis IHD					x			
Diagnosis Stroke/ TIA					x			
Diagnosis dizziness	x	x	x	x	x	x	x	x
Diagnosis Collapse		x	x	x	x	x	x	x
Systolic BP (mean)	x	x	x	x	x	x	x	x
Diastolic BP (mean)	x	x	x	x	x	x	x	x
Pulse rate (mean)				x	x			x
BMI								x
Ethnicity White Non-White	x	x	x	x	x		x	
Log mean pulse ^3,3	x	x	x		x	x	x	x
**AIC**	14806	14806	14804	14824	14779	14991	14895	14964
**BIC**	14907	14914	14920	14933	14950	15100	14996	15088
**AUC**	0.669	0.669	0.67	0.668	0.673	0.6401	0.66	0.6421
